# Clinical evaluation of rapid fluorescent diagnostic immunochromatographic test for influenza A virus (H1N1)

**DOI:** 10.1038/s41598-018-31786-8

**Published:** 2018-09-07

**Authors:** Seung-Taek Yu, Cuc Thi Bui, Do Thi Hoang Kim, Anh V. T. Nguyen, Thuy Tien Thi Trinh, Seon-Ju Yeo

**Affiliations:** 1 Department of Pediatrics, School of Medicine, Wonkwang University, 460, Iksan-daero, Iksan, 54538 Republic of Korea; 2 Zoonosis Research Center, Department of Infection Biology, School of Medicine, Wonkwang University, Iksan, 570-749 Republic of Korea

## Abstract

Rapid diagnostic tests (RDTs) have been developed to detect influenza A virus for the swift diagnosis and management of patients. However, despite the simplicity and convenience, the low sensitivity of RDTs remains a limitation for their use in point of care testing (POCT). In this study, we developed a rapid fluorescent immunochromatographic strip test (FICT) and the performance of FICT was confirmed by the real-time reverse transcription-polymerase chain reaction (rRT-PCR) of H1N1, compared with that of RDT. The limit of detection (LOD) of FICT was improved by 16-fold compared to RDT. FICT showed 85.29% sensitivity (29/34) (95% Confidence Interval [95% CI]: 68.94 to 95.05), 100% specificity (26/26) (95% CI: 86.78 to 100.00), and a strong correlation (kappa; 0. 92) compared with rRT-PCR (20 ≤ Ct ≤ 36). In contrast, RDT (Standard Diagnostics [SD] BIOLINE Influenza Ag A/ B/ A(H1N1) Pandemic) showed 55.88% sensitivity (19/34) (95% CI: 37.87 to 72.82), 100% specificity (26/26) (95% CI: 77.07 to 100), and had a fair correlation with rRT-PCR (kappa; 0. 75). FICT had better sensitivity than RDT (*P* < 0.01; McNemar’s test). Therefore, FICT has the potential to improve the quality of current rapid POCT for the diagnosis of influenza A/H1N1 infection.

## Introduction

Following early outbreaks in North America in April 2009, influenza A/H1N1 spread fast around the world by person to person contact prompting the World Health Organization (WHO) to declare a pandemic^1^. In August 2010, the pandemic ended with more than 18,449 deaths reported worldwide^2^. While H1N1 is not as alarming as it seemed a few years ago, it is still important to protect humans from H1N1 as it can cause serious health problems for some people^3^. Recently, 10,000 cases of pandemic H1N1 were reported of which 774 cases died^4^.

Appropriate treatment of patients with respiratory illness depends on the accurate and timely diagnosis^5^. Therefore, early and accurate diagnosis of influenza is expected to reduce the inappropriate use of antibiotics and provide the option of using antiviral therapy^6^.

During a respiratory illness outbreak in a closed setting (e.g., hospitals, long-term care facility, cruise ship, boarding school, and summer camp) testing for influenza can be very helpful in determining if it is the cause of the outbreak^7^. The accuracy of clinical diagnosis based on signs and symptoms alone is limited because symptoms from illness caused by other pathogens can overlap considerably with influenza^8^.

Diagnostic tests available for influenza include viral culture, serology, rapid antigen testing, real-time reverse transcription polymerase chain reaction (rRT-PCR), immunofluorescence assays, and rapid molecular assays^9^. Despite high sensitivity, rRT-PCR has a disadvantage as a point of care testing (POCT) as it requires professional expertise and specialized equipment^9^. Because antiviral drug should be administrated as early as possible after the onset of symptoms to be efficacious, rapid diagnosis of influenza infection is essential to help minimize the individual and economical loss^10^. Therefore, development of influenza rapid diagnostic tests (RDTs) would be important in controlling viral infection^10^. To date, more than 10 RDTs have been approved by the U.S. Food and Drug Administration (FDA)^11^. Most RDTs are read by naked eye, and some utilize an analyzer reader device to standardize result interpretation^11^. Sensitivity and specificity of these assays range between 50–70% and 90–95%, respectively and vary with the laboratory that performs the test, the type of test used, the time from the onset of illness to specimen collection, and the type of specimens tested^12^. For the diagnosis of seasonal influenza infections, RDTs have demonstrated variable performance with sensitivities ranging between 10%–70%, with up to 90% specificity compared to standard RT-PCR-based assays^13^. A meta-analysis reported pooled sensitivities for detecting influenza A as 54.4% for RDTs and 91.6% for rapid nucleic acid amplification tests (NAATs)^14^.

To enhance the sensitivity of RDT, the fluorescent material was employed in the rapid diagnostic system^15^. As a commercial fluorescent material, Europium nanoparticles (Eu NP) have been used to increase the sensitivity of immunoassays in ELISA^16^. In our previous study, Eu NP improved the sensitivity of conventional RDT to detect influenza A virus^17^.

However, sensitivity and specificity of Eu NP-based RDT for influenza A diagnosis in clinical specimens were not determined.

In this study, we developed a rapid fluorescent immunochromatographic strip test (FICT) employing Eu NP to improve the sensitivity of POCT for influenza A. The performance of FICT was confirmed by the real-time reverse transcription-polymerase chain reaction (rRT-PCR), and compared with that of a commercial RDT. Furthermore, the clinical performance of rapid FICT assay for influenza A was evaluated in H1N1-positive patients’ specimens.

## Materials and Method

### Reagents

Monoclonal antibodies (mAb), including anti-influenza A nucleoprotein (NP) (7307 and 7304), were purchased from Medix Biochemica (Espoo, Finland). Europium nanoparticles (Eu NP) (200 nm) were acquired from Bangs Laboratories Inc. (Fishers, IN, USA). N-(3-Dimethylaminopropyl)-N′-ethylcarbodiimide hydrochloride (EDC) and N-hydroxysulfosuccinimide sodium salt (Sulfo-NHS) were obtained from Thermo Scientific (Waltham, MA, USA). All other chemicals were purchased from Sigma-Aldrich (St. Louis, MO, USA). Influenza A/H1N1/2009/CA was provided by Korea Centers for Disease Control and Prevention. It was grown and titrated in eggs as previously described^18^. Influenza A/H3N2 isolated from human in 2014 was donated from Korean National Research Resource Center (Seoul, Korea) (registration number: KBPV-VR-85).

### Conjugation

The conjugation of the mAb to Eu NP was conducted by the EDC/NHS chemical reaction where NHS esters react with primary amines on the antibody to yield stable amide bonds^19^. Briefly, 10 µL of Eu NP (200 nm, 1% w/t) was added to 500 µL of 0.1 M Tris-HCl (pH 7.0) and incubated for 1 h at 25 °C in the presence of 0.13 mM EDC and 10 mM sulfo-NHS. Surplus EDC and sulfo-NHS were removed by centrifugation at 27, 237 × g for 5 min. The activated Eu NP was added to 30 µL of antibody (anti-influenza A NP) (1 mg/mL) in 500 µL of 0.1 M sodium phosphate (pH 8.0) and allowed to react for 30 min at 30 °C. After centrifugation at 27, 237 × g for 5 min, the Eu NP -conjugated antibody was collected, washed with phosphate-buffered saline (PBS) (pH 7.4), resuspended in 100 µL storage buffer (1% BSA in PBS), and stored at 4 °C.

### FICT and RDT

A test strip consisted of four components: sample application pad, conjugate pad, nitrocellulose membrane, and absorbent pad. The test line (TL) of the strip was prepared by dispensing the desired volume of 2.5 mg/mL mouse monoclonal antibody (anti-influenza A NP) and 0.5 mg/mL mouse IgG on the control line (CL).

To analyze the Eu NP-conjugated antibody-based diagnostic performance of FICT, 2 µL of Eu NP-conjugated antibody was applied onto the conjugate pad, and a mixture of 75 µL of samples and 75 µL of lysis buffer (100 mM Tris-HCl, pH 8.0, 0.025 M EDTA, 0.5% sodium deoxycholate, and 1% Triton X-100) were added to the sample pad for 15 mins.

Influenza A virus was reactive with the Eu NP-conjugated antibody, and the complexes were allowed to migrate to the immobilized antibody at TL. The fluorescent values were read by portable strip detector (Medisensor, Daegu, Republic of Korea) (excitation 355 nm/emission 612 nm)^20^.

As RDT, Standard Diagnostics (SD) BIOLINE Influenza Ag A/B/A(H1N1) (Yongin, Republic of Korea) was compared with FICT. The same volume of nasopharyngeal samples and virus diluent were tested, and all procedures were performed according to the instructions provided by the manufacturer.

### Real-time RT-PCR (rRT-PCR)

rRT-PCR was performed using a Probe RT-PCR Kit (QIAGEN, Hilden, Germany) and the cycle threshold (Ct) values were determined using a CFX96 Real-Time PCR Detection System (Bio-Rad, Hercules, CA, USA). The hemagglutinin 1 (HA1) primers, probes, and RT- PCR conditions have been described previously^21^. For the standard of RNA copy number, the template was generated in plasmid pGEM-T Easy (Promega, Madison, WI, USA) including a 187-base pair (bp) insert. RiboMax (Promega) kit was used for the *in vitro* transcription of HA1 RNA and the RNA copy number for the limit of detection of FICT was determined. The standard curve was calculated automatically by plotting the Ct values against each standard of known RNA copy number and by extrapolating the linear regression line of this curve.

### Biosafety

As influenza A/H1N1/2009/CA is classified as a biosafety level-2 (BSL-2) pathogen by WHO, all studies were conducted at class II biosafety cabinet in a BSL-2 laboratory. Influenza A/H1N1/2009/CA was inactivated by temperatures above 56 °C for at least 30 min following a previous report^22^.

### Ethical statement

The study was approved by the Institutional Review Board of the Wonkwang University Hospital (Approval No. 1263), and all methods were carried out in accordance with relevant guidelines and regulations. All patients agreed to participate in this study, and informed consent was processed before taking specimens.

### Statistics

The mean ± standard deviation (SD) was calculated, and all data were plotted using GraphPad Prism 5.0 (GraphPad, La Jolla, CA, USA). Statistical analysis of the sensitivity and specificity with 95% Confidence Interval (95% CI) of each assay was performed using MedCalc Statistical Software (version 12.3.0, MedCalc Software, Mariakerke, Belgium). McNemar’s test was used to determine the statistical differences between assays^23^.

## Results

### Working principle of the fluorescent diagnostic assays

**Figure 1:**
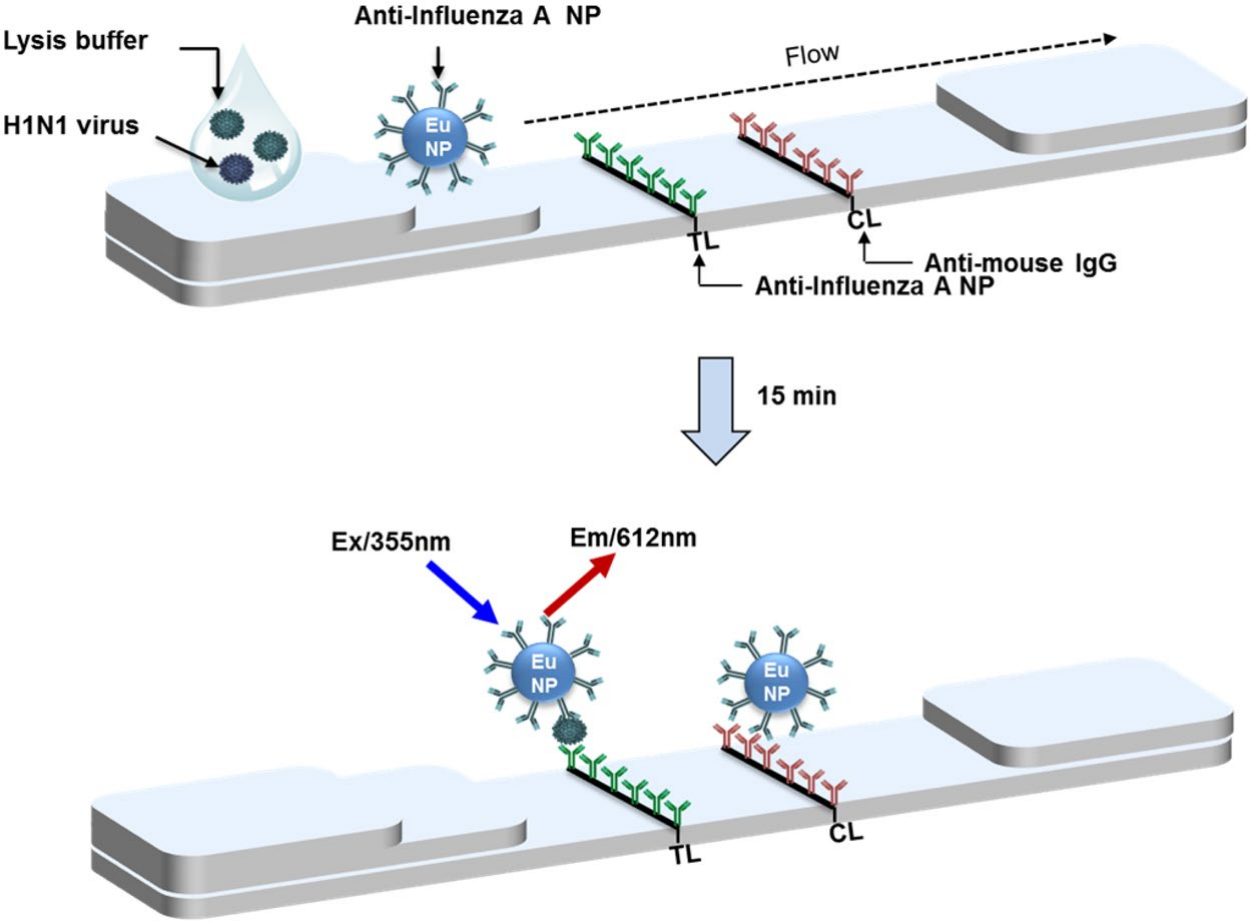
Schematic diagram of Eu NP-Ab conjugate-linked rapid fluorescent immunochromatographic strip test (FICT). Fluorescent diagnostic system using the conjugate of the antibody to Europium nanoparticles (Eu NP) works with 75 μL of specimen with 75 μL of lysis buffer on strip coated with anti-influenza A nucleoprotein (NP) at test line (TL) and anti-mouse IgG at control line (CL). Each fluorescence intensity is measured within 15 minutes by the portable fluorescent detector. The excitation and emission wavelengths measured were 355 nm and 612 nm, respectively. TL/CL value was used for quantitative diagnostic values of FICt assay.

Eu NP-conjugated antibody was prepared by conjugation of antibody on Eu NP to form an amide bond. As shown in Fig. 1, Eu NP-conjugated antibody was applied to the conjugate pad of the diagnostic strip. Subsequently, 75 µL of the specimen was added on to the sample pad followed by 75 µL of lysis buffer to expose antigen from virus and complete the lateral flow reaction. After allowing 15 min for the completion of the reaction, the kit was read using the portable fluorescence detector. The excitation and detection wavelengths of Eu NP were 355 nm and 612 nm, respectively. The portable fluorescent detector displays the fluorescence intensities at test line (TL) and control line (CL) which is used for determination of TL/CL ratio.

### The limit of detection (LOD) of assays

**Figure 2:**
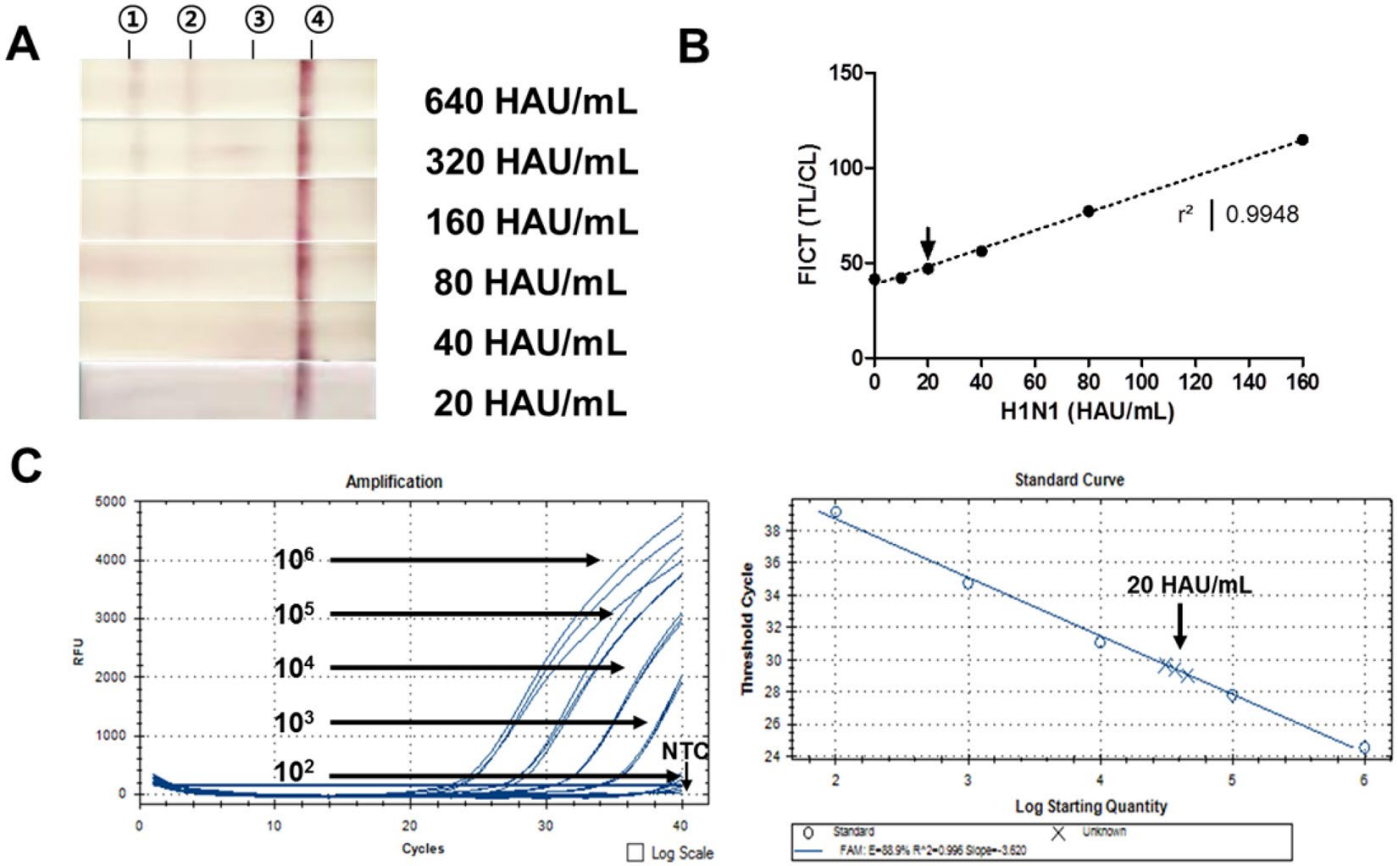
The limit of detection (LOD) of assays. (**A**) The performance of rapid diagnostic kit (RDT). After preparing two-fold dilutions of samples ranging from 20 HA/mL to 320 HAU/mL titers, 75 μL of the virus diluent was tested for 20 min. (**B**) The performance of FICT employing Eu NP. Fluorescent intensities of TL and CL on strips were captured at different H1N1 virus after application of two-fold dilutions of samples ranging from 10 HAU/mL to 160 HAU/mL titers. Data (*n* = 3) are shown as mean ± SD. (**C**) Determination of RNA copy number and threshold cycle (Ct) of LOD of FICT. The virus titer corresponding to LOD of FICT was subjected to RNA extraction and used for rRT-PCR. The linear relationship between Ct value and RNA copy number is shown in right panel. Arrow indicates the virus titer of LOD of FICT. NTC, no template control; ①, H1N1; ②, Influenza A; ③, Influenza B; ④, Control line.

After preparing two-fold dilutions of samples ranging from 20 HAU/mL to 640 HAU/mL, 75 μL of sample was tested in both FICT and commercial RDT assays. In RDT, limit of detection (LOD) was determined to be 320 HAU/mL because of the very faint band at test line **(**Fig. 2A). In FICT, the LOD was determined by the limit of blank (LOB)^24^. LOD was the lowest analytic concentration likely to be reliably distinguished from the LOB at which detection was feasible. LOB and LOB formulae used were mean (blank) + 1.645 (standard deviation (SD) of blank) and LOB + 1.645 (SD of low concentration sample), respectively. Accordingly, LOB of FICT was 43.18 and LOD was 44.20, which was below the value of 20 HAU/mL (TL/CL: 46.91 ± 0.61) for the H1N1 virus **(**Fig. 2B**)**. Therefore, LOD of FICT was determined to be 20 HAU/mL Figs S1 and S2 display the raw RDT- and FICT results, respectively. We also determined the RNA copy number of the LOD (20 HAU/mL of the H1N1 virus) of FICT. With 20 HAU/mL of the virus, 75 μL of sample was used for RNA extraction and the eluted RNA was used for rRT-PCR. To generate a calibration curve, serially-diluted RNA standard was used (Fig. 2C). A standard curve was drawn to show the starting copy number of the standard RNA vs the cycle threshold (Ct). The plot of a standard curve of Ct values against the logarithmic dilutions produced an r^2^ value >0. 99 and the slope corresponded to efficiency in the range 88.9%, which was close to the optimized protocol. rRT-PCR data of RNA standard was presented in Fig. S3. The 20 HAU/mL of H1N1 virus showed a Ct value of 28.62 ± 0.51 (mean ± SD), and the RNA copy number of FICT LOD was 4.59 × 10^4^ ± 18,060 (mean ± SD).

### Characterization of patient population

Nasopharyngeal specimens from influenza A-confirmed patients listed in Wonkwang Hospital, Iksan between 2012 and 2014 were tested by FICT. A total of 62 patients were included in the analysis whose general characteristics are presented in Table 1. There were more male than female patients with average age of 13 years. Specimens were collected within one week after onset of symptoms. One patient in the study had *Streptococcus pneumoniae* (*n* = 1). Specimens from patients with the common viral respiratory disease, human rhinovirus (HRV) (*n* = 4) and influenza B (*n* = 4) were also included. Among H1N1- negative cases by rRT-PCR, two specimens were included as non-H1N1 subtype of influenza A because they were strongly positive in influenza A RDT in addition to patient record, implying that it may not be H1N1 infection but another subtype of influenza A.

**Table 1:** Characteristics of the patients.

Variables	Number
Total patients	62
Age, years, mean (SD)	13 (11)
Sex (Male:Female)	38:27
Mean of date of specimen collection after onset of symptom (range)	4 (3–5)
Seasonal period	2012–2014

### Clinical performance of assays

As shown in Table 2, all Ct values in the negative group by rRT-PCR using H1N1 probe/primers were higher than 36.4. The presence of RNA in each sample was confirmed by gel electrophoresis. Only H1N1-positive samples showed 187 nucleotides in agarose gels (Fig. S3). Thirty-four patients were confirmed as H1N1-rRT-PCR positive, showing the Ct value below 36 (Table 3). All values of rRT-PCR, RDT, and FICT are summarized in Tables 2 and 3. *Streptococcus pneumoniae* (*n* = 1) -, human rhinovirus (HRV) (*n* = 4) -, and influenza B (*n* = 4) - infected patients showed negative values in FICT assay.

**Table 2:** Comparison of rRT-PCR, RDT, and FICT in clinical specimens of H1N1-rRT-PCR negative patients.

#	Specimen #	Age(yr)	Sex	Disease	Ct^b^ value	H1N1 RNA copy number/reaction	Influenza A RDT^c^	Influenza A FICT	
								TL/CL	Result
1	N.1	6	M	Flu A	37.87	708.34	N	15.95	N
2	N.2	2	F	Flu A	37.35	949.27	N	17.82	N
3	N.6	4	F	Flu A	39.62	263.48	N	20.71	N
4	N.7	7	M	Flu A	37.81	732.17	N	11.78	N
5	N.17	3	F	Flu A	37.32	965.05	N	13.65	N
6	N.23	11	M	Flu A	37.28	986.94	N	14.29	N
7	N.26	5	M	Flu A	37.95	677.75	N	14.47	N
8	N.29	7	M	Flu A	38.24	575.61	N	18.25	N
9	N.30	6	M	Flu A	38.13	611.36	N	20.74	N
10	N.33	7	M	Flu A	38.16	602.14	N	21.37	N
11	N.34	5	F	Flu A	37.96	672.49	N	16.84	N
12	N.36	8	F	Flu A	37.82	728.2	N	20.40	N
13	N.37	6	M	Flu A	37.62	814.01	N	16.47	N
14	N.38	12	F	Flu A	37.91	690.61	N	22.20	N
15	N.39	12	M	Flu A	37.72	772.86	N	17.35	N
16	N.40	3	M	Flu A	37.92	686.66	N	4.94	N
17	N.42	7	M	Flu A	36.48	1552.63	N	19.59	N
18	S.pneu 1.2	2	M	*Staphylococcus pneumoniae*	0.00	0.00	N	11.62	N
19	HRV 1.6	10	F	HRV^a^	0.00	0.00	N	18.46	N
20	HRV 2.7	8	M	HRV	0.00	0.00	N	21.28	N
21	HRV 15.5	13	M	HRV	0.00	0.00	N	15.83	N
22	HRV 17.6	3	M	HRV	0.00	0.00	N	20.08	N
23	Flu B.47.10	34	M	Flu B	0.00	0.00	N	20.43	N
24	Flu B.47.28	17	F	Flu B	0.00	0.00	N	20.97	N
25	Flu B P20.10	7	M	Flu B	0.00	0.00	N	18.37	N
26	Flu B P24.15	12	F	Flu B	0.00	0.00	N	17.84	N

^a^HRV: Human rhinovirus.

^b^Ct: Cycle threshold of H1N1 rRT-PCR.

^c^RDT: SD BIOLINE Influenza Ag A/ B/ A(H1N1).

**Table 3:** Comparison of rRT-PCR, RDT, and FICT in clinical specimens of H1N1-rRT-PCR positive patients.

#	Specimen #	Age(yr)	Sex	Disease	Ct value	H1N1 RNA copy number/reaction	Influenza A RDT	Influenza A FICT	
								TL/CL	Result
1	P.3	12	M	Flu A	28.52	181598.91	N	49.82	P
2	P.4	7	F	Flu A	21.17	11598488.42	P	70.74	P
3	P.5	9	F	Flu A	25.54	983916.13	N	26.89	P
4	P.9	5	F	Flu A	22.77	3666082.66	P	34.24	P
5	P.10	17	F	Flu A	25.28	883630.4	N	23.05	P
6	P.11	13	M	Flu A	22.05	5523812.72	P	95.63	P
7	P.12	23	F	Flu A	27.02	330842.51	N	21.92	N
8	P.14	36	M	Flu A	31.31	29079.41	N	33.30	P
9	P.15	12	F	Flu A	24.34	1511498.4	N	18.19	N
10	P.16	4	F	Flu A	27.51	250527.41	N	26.14	P
11	P.18	6	M	Flu A	22.36	4636754.01	P	34.61	P
12	P.20	5	M	Flu A	27.69	291702.35	N	26.96	P
13	P.21	2	M	Flu A	29.14	99673.57	N	35.09	P
14	P.22	9	M	Flu A	29.98	79623.89	P	71.52	P
15	P.25	10	M	Flu A	21.37	8106853.83	P	34.03	P
16	P.28	54	F	Flu A	26.23	516059.05	N	12.52	N
17	P.35	17	M	Flu A	27.18	388044.67	N	15.59	N
18	P.36	13	F	Flu A	23.99	2364216.85	P	73.74	P
19	P.39	52	M	Flu A	31.3	37887.15	P	14.28	N
20	P.42	14	M	Flu A	27.39	345279.57	N	24.58	P
21	P.45	2	M	Flu A	21.25	11119361.9	N	27.30	P
22	P.48	4	F	Flu A	30.7	53193.44	P	23.11	P
23	P.104	27	M	Flu A	31.78	22239.68	P	23.10	P
24	P.169	23	F	Flu A	29.47	82734.83	N	47.92	P
25	P.188	6	M	Flu A	28.02	187696.44	N	23.83	P
26	P.190	23	M	Flu A	23.2	2882944.28	P	27.12	P
27	P.197	33	F	Flu A	29.61	76220.98	P	66.35	P
28	P.225	35	M	Flu A	25.09	986930.18	P	52.34	P
29	K.14.1	22	F	Flu A	27.59	224324.45670	P	50.51	P
30	K.33.2	7	M	Flu A	26.53	420871.72638	P	74.06	P
31	K.72.1	19	M	Flu A	24.38	1507210.69605	P	88.24	P
32	K.24.2	8	F	Flu A	22.07	5903487.54936	P	39.50	P
33	K.27.1	12	F	Flu A	25.44	804038.51737	P	36.68	P
34	K.63.1	32	M	Flu A	23.39	2703335.40786	P	61.95	P

The sensitivity of the new FICT assay was compared to that of a standard rRT-PCR for the H1N1 gene of influenza virus A. To evaluate the correlation between rRT-PCR and the FICT assay, kappa statistic was employed as described in a previous report^25^. RDT and FICT showed 46.15% (6/13) (95% confidence interval [CI]: 19.22 to 74.87) and 84.61% (11/13) (95% CI: 44.90 to 92.21) results with samples having Ct values between 20 and 25 (Table 4). In the range of 25 ≤ Ct ≤ 36, RDT could detect the antigen in 13 out of 21 patients (61.90%; 95% CI: 38.44 to 81.89) whereas 18 out of 21 patients were positive by FICT (85.71%; 95% CI: 63.66 to 96.95). Therefore, compared to rRT-PCR, FICT showed 85.29% sensitivity (29/34) (95% CI: 68.94 to 95.05) (20 ≤ Ct ≤ 36) and had a strong correlation (kappa; 0. 92). In contrast, RDT showed 55.88% sensitivity (19/34) (95% CI: 37.87 to 72.82) and had a fair correlation with rRT-PCR (kappa; 0. 75) (Table 5). Among all H1N1-rRT-PCR negative specimens (*n* = 28), two specimens were positive by influenza A according to patient information and two assays (RDT and FICT), indicating that those samples could be infected with non-H1N1 subtype of influenza A. Therefore, those two influenza A-positive and RDT positive patients were excluded from the influenza A negative group and only 26 patients were used for determining the specificity (*n* = 26). Those specimens presented the TL/CL ratio lower than the cut-off value of TL/CL (22.5), indicating that FICT had 100% specificity (26/26) (95% CI: 86.78 to 100.00). RDT also showed 100% specificity (26/26) (95% CI: 77.07 to 100). To compare the FICT with RDT, McNemar’s test was conducted and the *P* value was calculated using the MedCalc statistical software. As shown in Table 6, FICT showed better sensitivity than RDT by McNemar’s test (*P* value: 0.0063).

**Table 4:** Clinical diagnostic performance of FICT assay.

H1N1- rRT-PCR	Ct	Commercial RDT		FICT	
		Sensitivity	Specificity	Sensitivity	Specificity
Positive (*n* = 34^a^)	20 ≤ Ct < 25	46.15% (6/13)		84.61% (11/13)	
	25 ≤ Ct ≤ 36	61.90% (13/21)		85.71% (18/21)	
	20 ≤ Ct ≤ 36	55.88% (19/34)		85.29% (29/34)	
Negative (*n* = 26^b^)	Ct > 36		100% (26/26)		100% (26/26)

^a^Specimens #1 to #34 among H1N1-rRT-PCR positive cases were used to determine the sensitivity of assays.

^b^Specimen #1 to #26 among H1N1-rRT-PCR negative cases were used to determine the specificity of assays.

**Table 5:** Comparison of the clinical diagnostic performance of FICT assay with rRT-PCR and commercial RDT.

		Commercial RDT			FICT		
		Positive	Negative	Row Marginal	Positive	Negative	Row Marginal
H1N1 rRT-PCR	Positive^a^	19	15	34	29	5	34
	Negative^b^	0	26	26	0	26	26
Column Marginal		19	41	60	29	31	60
% Agreement (kappa)		0.75 [(19 + 26)/60]			0.92 [(29 + 26)/60]

**Table 6:** Comparison of the clinical diagnostic performance of FICT assay with commercial RDT by McNemar’s test.

		Commercial RDT		
		Positive	Negative	Row Marginal
FICT	Positive	18	11	29
	Negative	1	4	5
Column Marginal		19	15	34

**Figure 3:**
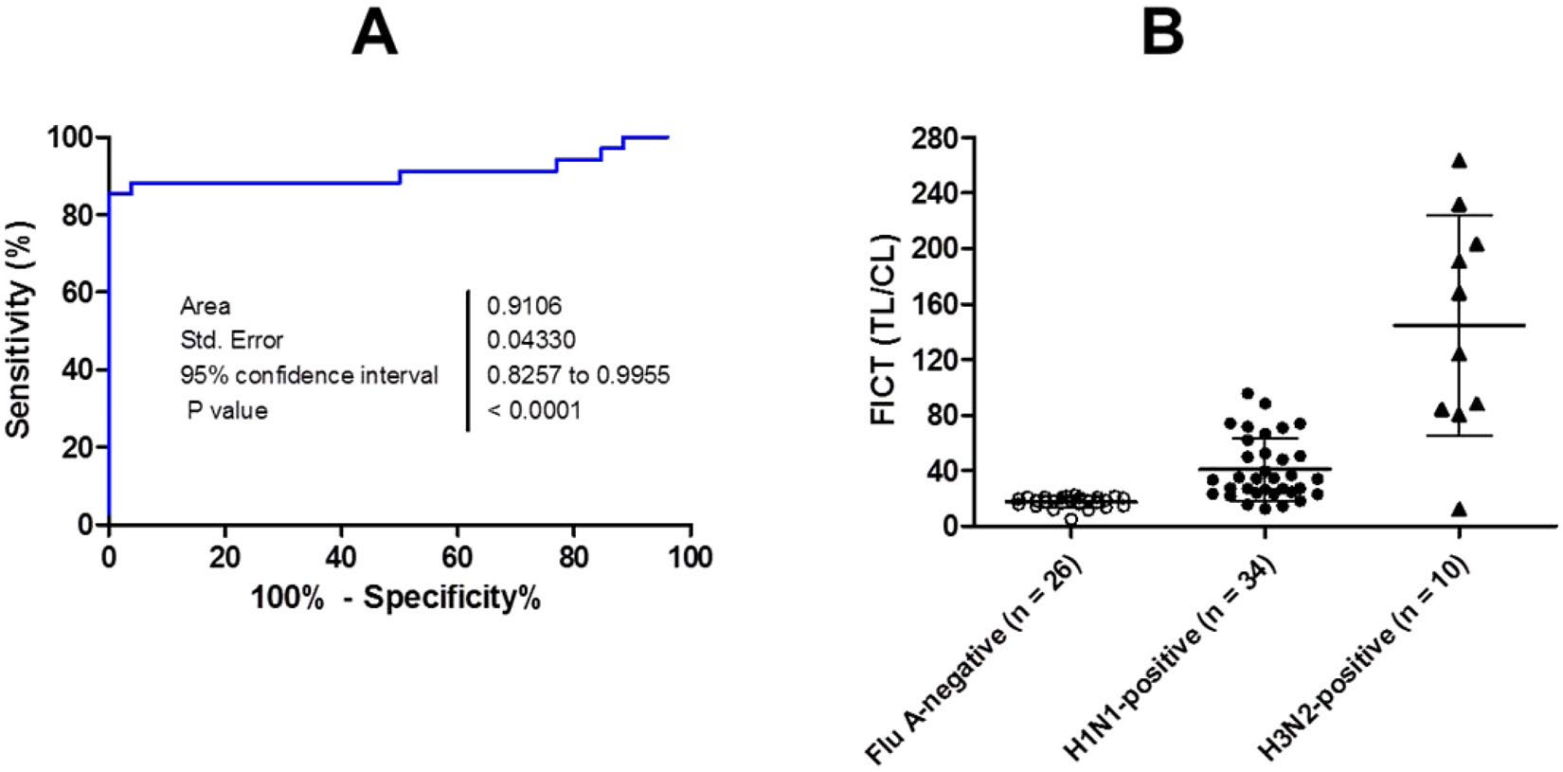
Analysis of FICT assay with patient specimens. (**A**) The accuracy of FICT performance was determined with the area under the ROC curve (AUC) by testing negative and positive patients for H1N1 rRT-PCR. (**B**) Based on the ROC curve analysis, 22.5 was determined as the threshold cut-off value to differentiate H1N1 infection. H3N2-positive patients (*n* = 10) were additionally tested in FICT. All fluorescent levels of TL/CL were plotted to predict influenza A infection.

The TL/CL threshold cut-off value for H1N1 was determined to be 22.5 from receiver-operating characteristic (ROC) curve analysis after plotting all data using GraphPad Prism (positive if TL/CL >22.5, negative otherwise). According to the ROC curve analysis, an area under-the-curve (AUC) value was 0.91 (95% CI: 0.83 to 0.99) for H1N1 patients (Fig. 3A) (P < 0.0001), indicating the high accuracy of FICT to predict influenza A infection for H1N1 infected patients. Among H1N1-negative patients (*n* = 26), none of the cases showed a false-positive TL/CL value. The H1N1-infected control group (*n* = 34) showed only 29 patients with TL/CL than the cut-off value (Fig. 3B).

Raw FICT and RDT values of clinical specimens are shown in Figs S4–S6. We used SD RDT which could indicate both influenza A- and H1N subtype information. The rRT-PCR result confirmed H1N1 infection in the specimens. Our results clearly showed that FICT could detect the pandemic strain of H1N1 as well as other subtypes of influenza A in the specimens. Additionally, the performance of FICT to detect H3N2 was confirmed (Fig. S7). To evaluate the diagnostic accuracy of FICT for H3N2-infected sample, ten clinical patients with 22.27 ≤ Ct ≤ 37.10 (1.39 × 10^2^ − 2.03 × 10^6^ of RNA copy numbers/reaction) were tested in FICT and nine out of ten influenza A (H3N2)-positive patients had higher TL/CL than the cut-off value in FICT assay (sensitivity of 90%), indicating the proposed diagnostic system had potential to predict H3N2 infection in clinical samples. Result of H3N2-rRT-PCR of clinical samples and FICT was shown in Figs S8 and S9, respectively.

## Discussion

Symptoms of influenza virus infection vary from an asymptomatic and uncomplicated upper respiratory tract infection to a dangerous condition of severe pneumonia with severe organ damage associated with exacerbation of the underlying disease^26^. Clinicians need fast assays to diagnose and manage the infection, especially during a high prevalence period^9^. It is, therefore, highly desirable to have access to POCT for rapid and reliable diagnosis. Several influenza RDTs have been developed for this purpose and are commercially available.

As influenza RDTs are developed to provide more sensitive and specific performance, newly developed technology as POCT requires test evaluation with conventional RDTs^27^. FICT assay used in this study had the same principle as RDT except for application of Eu NP as an indicator instead of colloidal gold in RDT and thus, the performance of FICT assay was assessed by RDT.

When the accuracy of the rapid diagnostic tests for H1N1 patients was compared to a reference test (“gold standard” = RT-PCR or viral culture), WHO reported the performance of the commercialized RDT as 23–26 of Ct values^28^. Therefore, a more sensitive and rapid diagnostic system would be preferable if it could efficiently detect the virus at 25 > Ct values.

In this study, we evaluated a novel fluorescent diagnostic system and compared it with the gold standard diagnostic method rRT-PCR as well as one RDT. In the range of 25 ≤ Ct ≤ 36, the performance of FICT employing Eu NP fluorescent material had 85.29% sensitivity which represents about 30% improvement relative to the commercialized RDT.

Although FICT was compared with rRT-PCR of H1N1 gene, this assay, in principle, is capable of detecting other subtypes of influenza A because the antibody used was against the influenza A nucleoprotein (NP). In our study, two influenza A (non-H1N1) were tested positive implying that FICT assay could be capable of detecting influenza A other subtypes.

A variety of simplified methods are being developed that can circumvent laboratory testing providing fast and reliable results at the POCT^29^. There are two categories of POCT technologies: the first one uses small hand-held devices whereas the second one often uses large bench-top devices which are essentially laboratory instruments which have been reduced in both size and complexity converting them into portable devices that can be used by non-technical personnel. One such portable fluorescent strip detector was used in our study and, as is shown in the schematic diagram in Fig. 1, could measure the fluorescent intensity in a point of care setting. To make the new FICT assay further suitable for POCT, sample treatment should be conducted in a dropper. Also, the data management of FICT can be further simplified by setting up a cut-off value that would be convenient for the doctors and shorten the time for decision-making and treatment.

In the future, limitation of small sample size used in this study will be addressed by analyzing a larger number of clinical specimens expected to provide greater significance. Although we compared FICT with a widely used commercial RDT, the sensitivity of the assay can be further strengthened by comparing it with multiple RDTs.

In conclusion, the newly developed FICT assay to diagnose influenza A infection showed a better performance than the SD RDT and a comparable performance to that of rRT-PCR. These data would aid in further developing and/or optimizing the newly developed Eu NP-linked rapid diagnostic system for other influenza A subtypes or influenza B type infection.

## Electronic supplementary material

Supplementary information
