# Supplementary material for: Clinical evaluation of rapid fluorescent diagnostic immunochromatographic test for influenza A virus (H1N1)
Source: Sci Rep. 2018 Sep 7;8:13468. doi: 10.1038/s41598-018-31786-8 (PMC6128899; doi:10.1038/s41598-018-31786-8)
Supplement: Supplementary file 1 — Supplementary information [file 41598_2018_31786_MOESM1_ESM.docx]

**Supplementary information**

**Clinical evaluation of rapid fluorescent diagnostic immunochromatographic test for influenza A virus (H1N1)**

Seung-Taek Yu^1‡^, Bui Thi Cuc^2‡^, Do Thi Hoang Kim^2‡^, Nguyen Thi Viet Anh^1^, Trinh Thi Thuy Tien^1^,Seon-Ju Yeo^2^**^§^**

**Supplementary Figures**

**Figure S1.** Raw results of Quantitative analysis by RDT (Figure 2A)

**Figure S2.** Raw results of Quantitative analysis by rapid FICT (Figure 2B)

**Figure S3.** Electrophoresis of rRT-PCR results of H1N-negative and –positive patients.

**Figure S4.** Raw results of rapid FICT of H1N1-negative clinical specimens

**Figure S5.** Raw results of rapid FICT of H1N1-positive clinical specimens

**Figure S6.** Raw results of RDT of clinical specimens

**Figure S7.** Performance of FICT to detect H3N2 virus.

**Figure S8.** rRT-PCR of H3N2-positive patients.

**Figure S9.** Raw results of FICT of H3N2-positive clinical specimens.

**
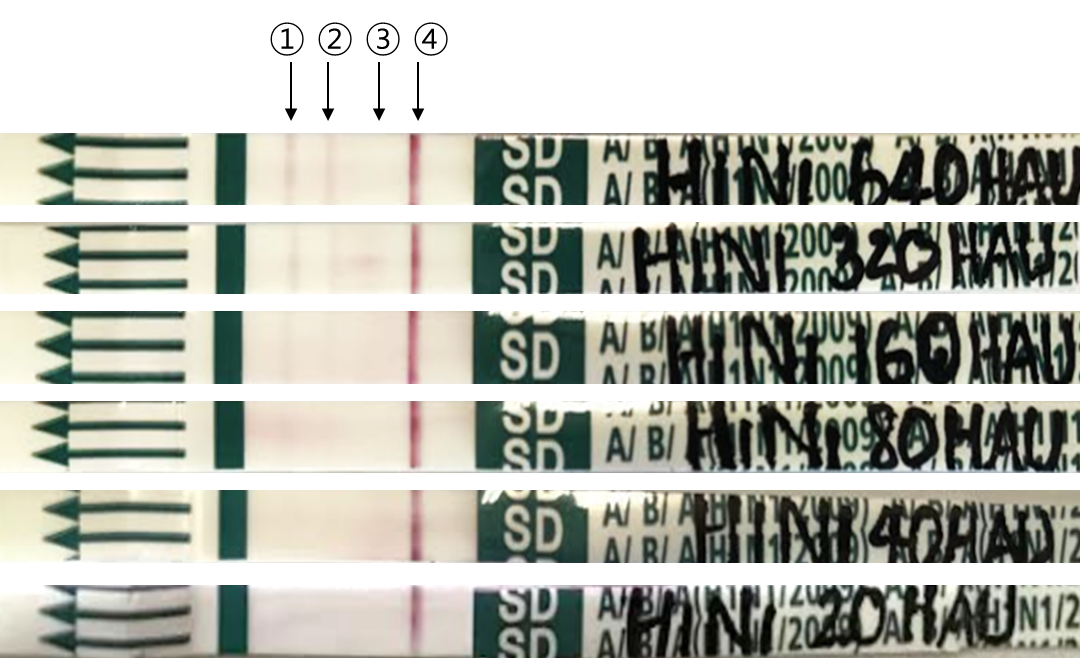
**

**Figure S1.** Raw results of Quantitative analysis by RDT (Figure 2A). Faint colors in test line of H1N1 and influenza A were detected with H1N1 virus (320 HAU/mL). ①, H1N1; ②, Influenza A; ③, Influenza B; ④, Control line

**
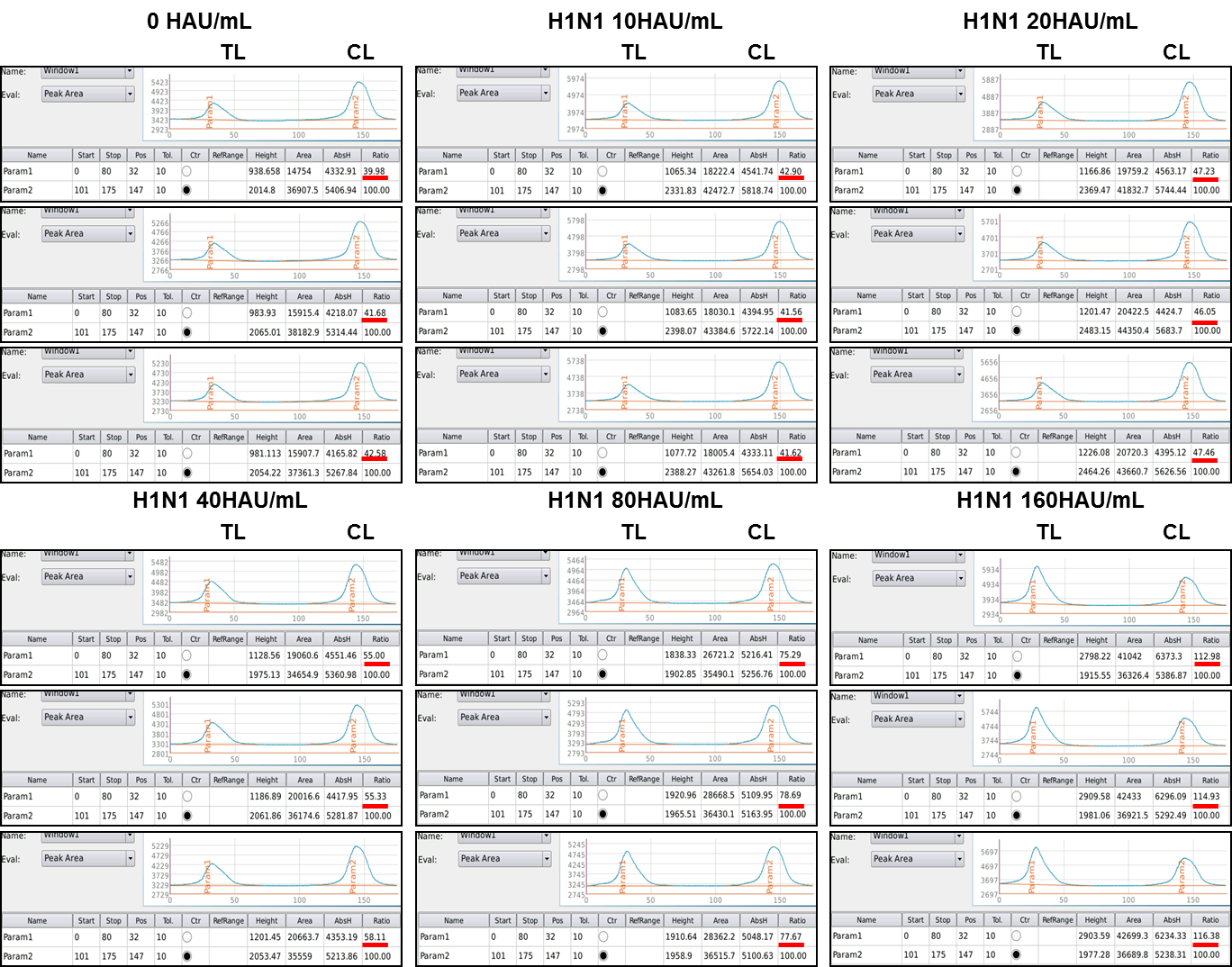
**

**Figure S2.** Raw results of Quantitative analysis by rapid FICT (Figure 2B). Each ratio of TL/CL was underlined with red bar (-).

**
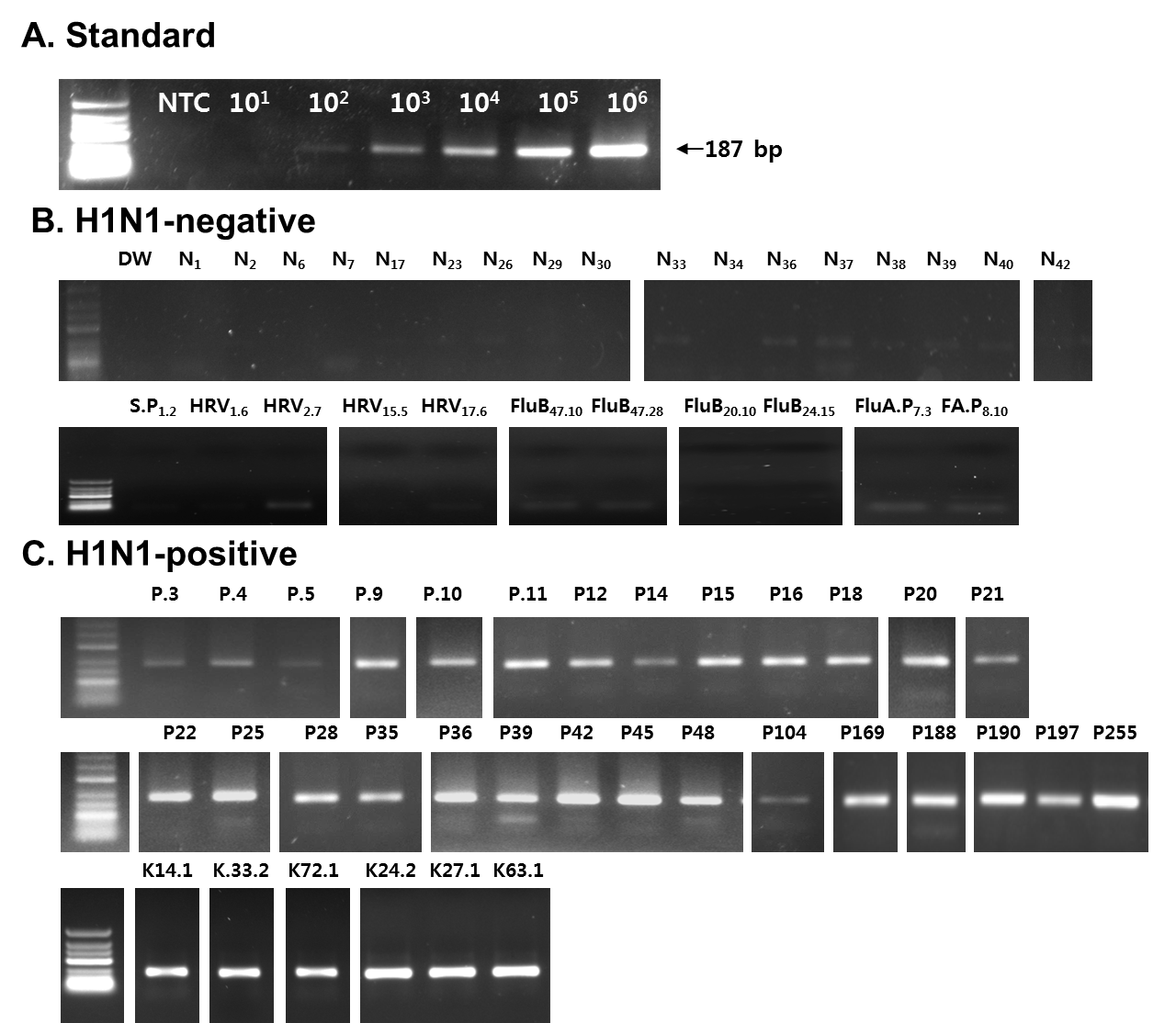
**

**Figure S3.** Electrophoresis of rRT-PCR result of standard, H1N1 – negative, and – positive samples. Experiments of rRT-PCR with clinical samples were performed and each sample was conducted in FICT- and RDT assay.

**
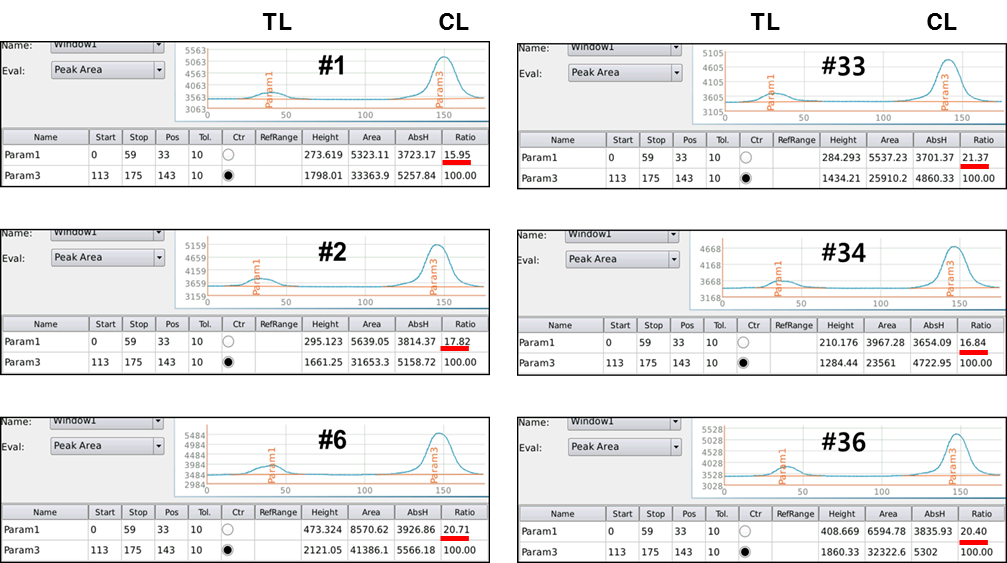
**

**
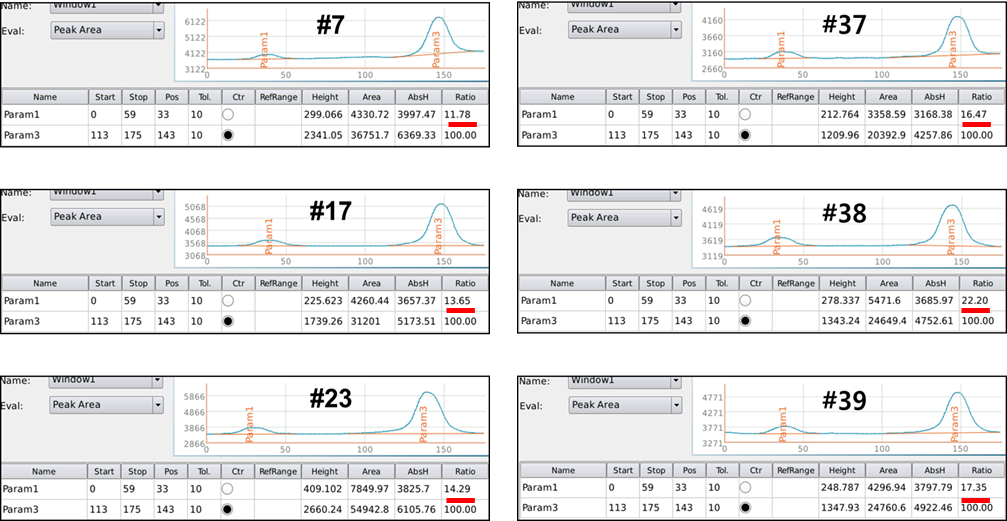
**

**
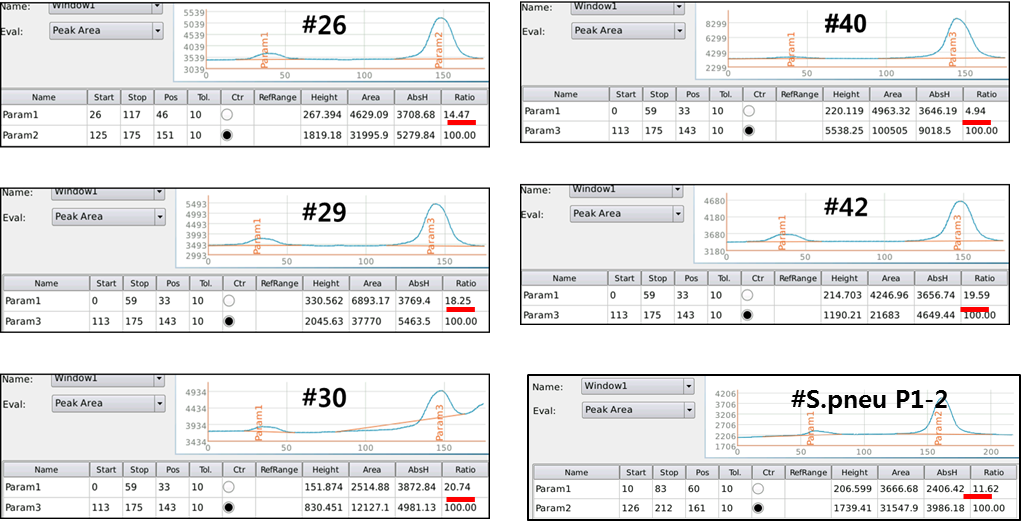
**

**
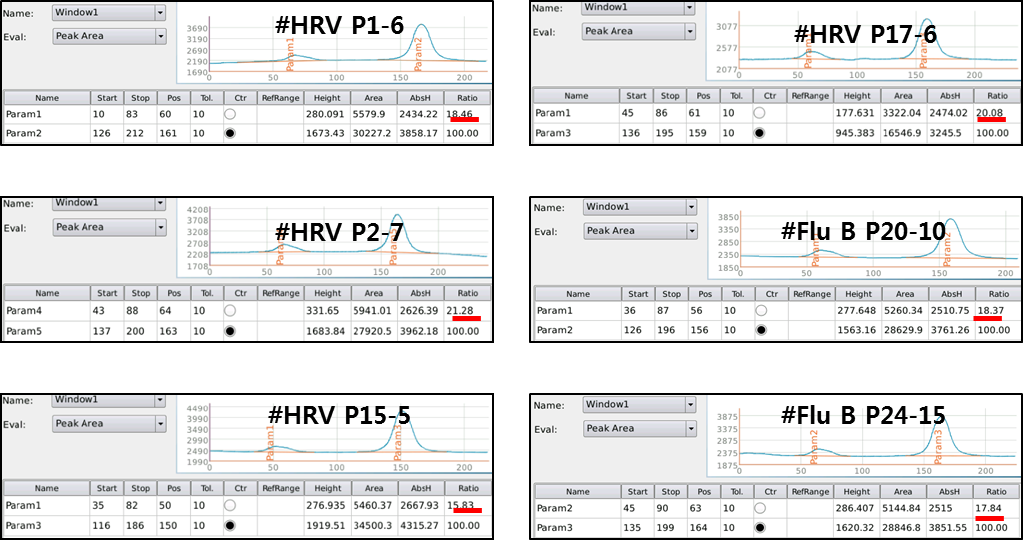
**

**
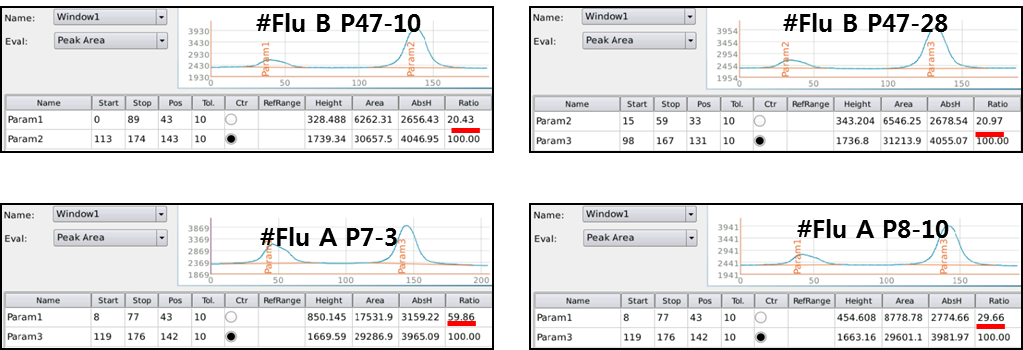
**

**Figure S4.** Raw results of rapid FICT of H1N1-negative clinical specimens. Each ratio of TL/CL was underlined with red bar (-).

**
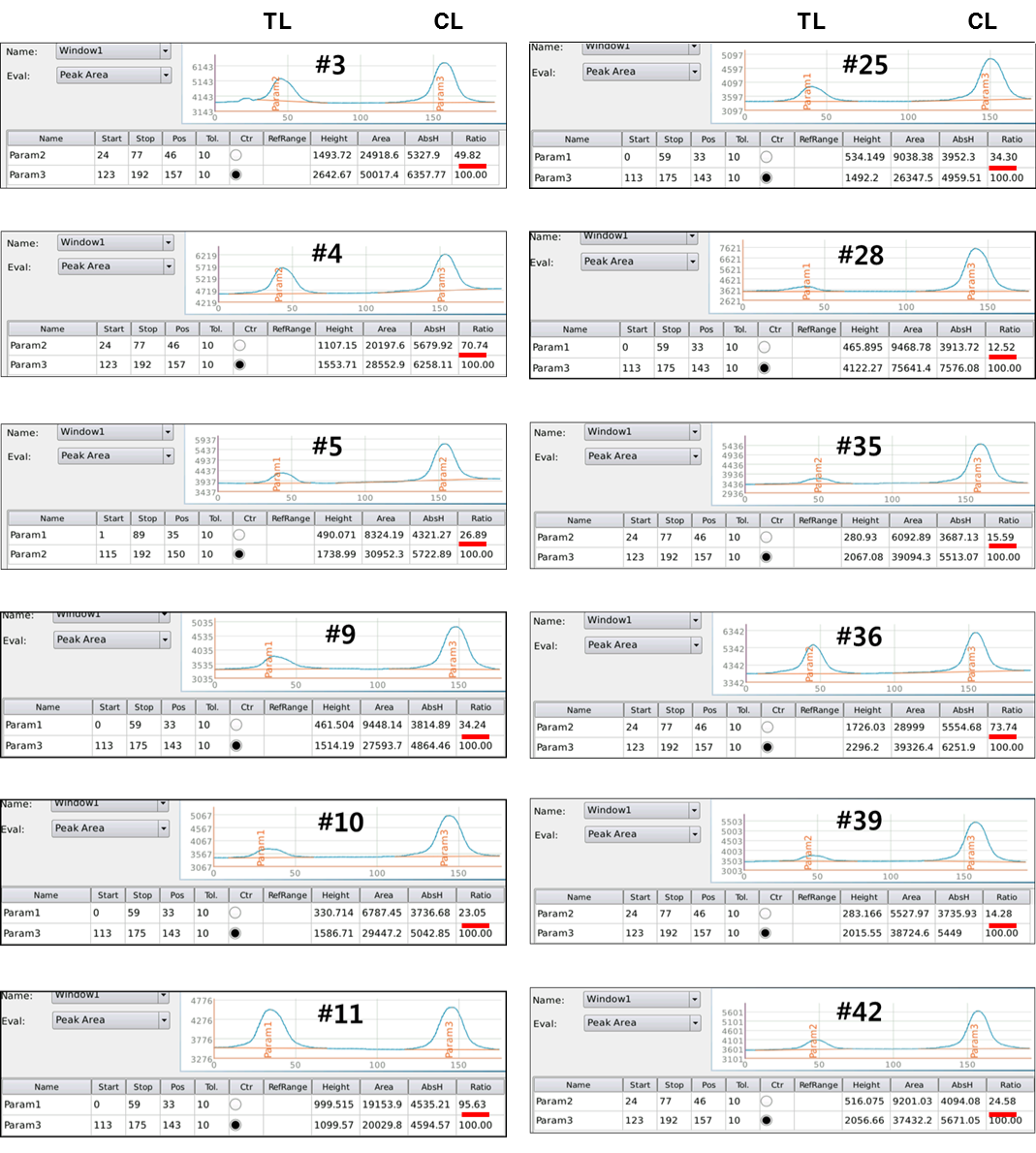

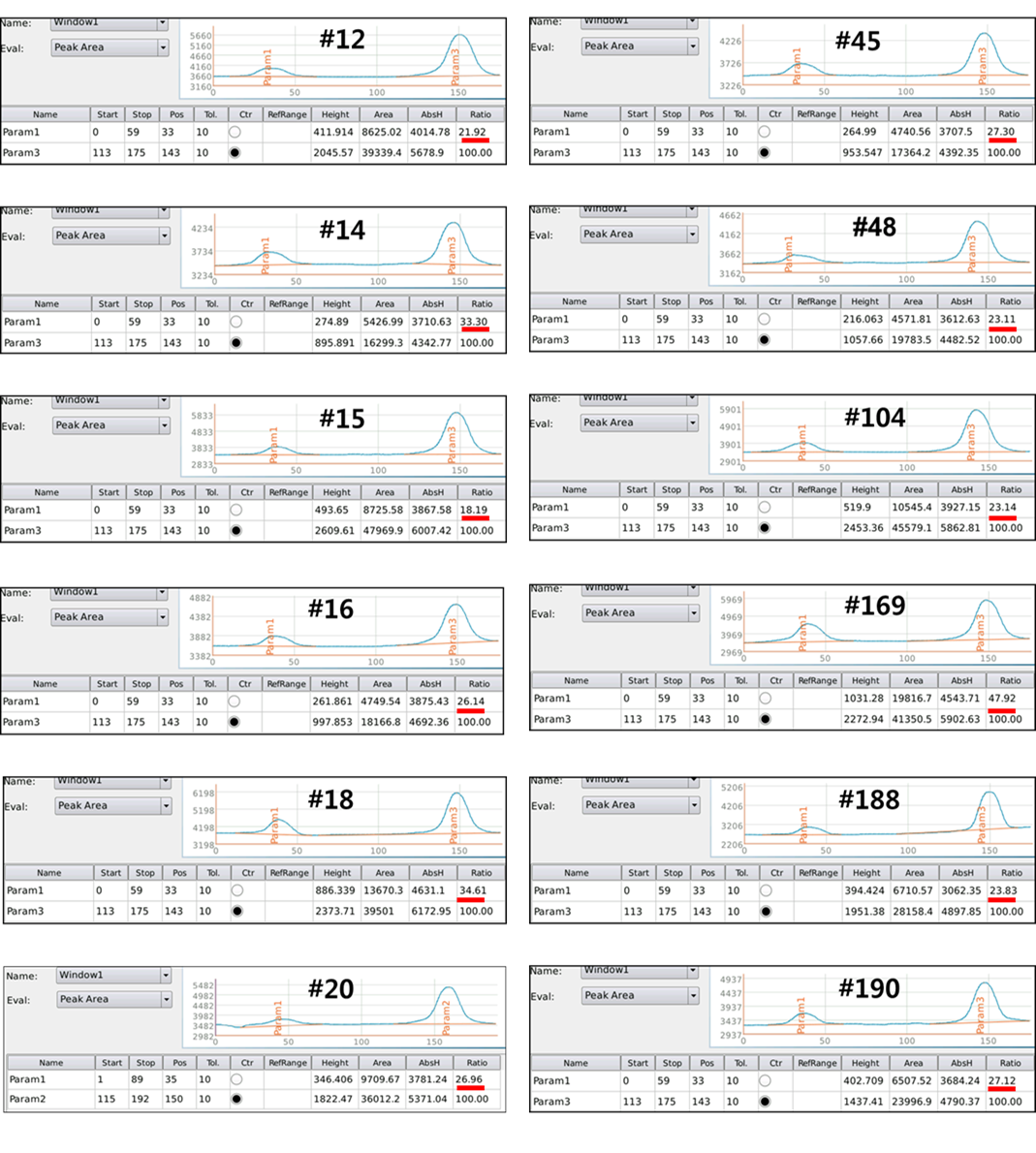
**

**
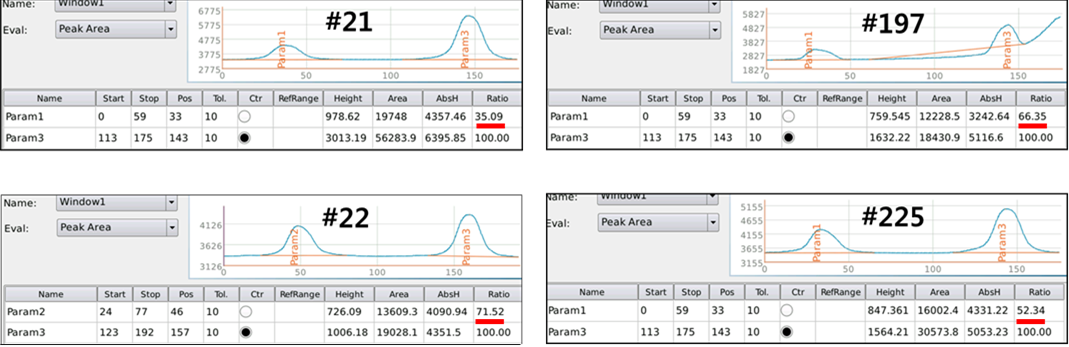
**

**
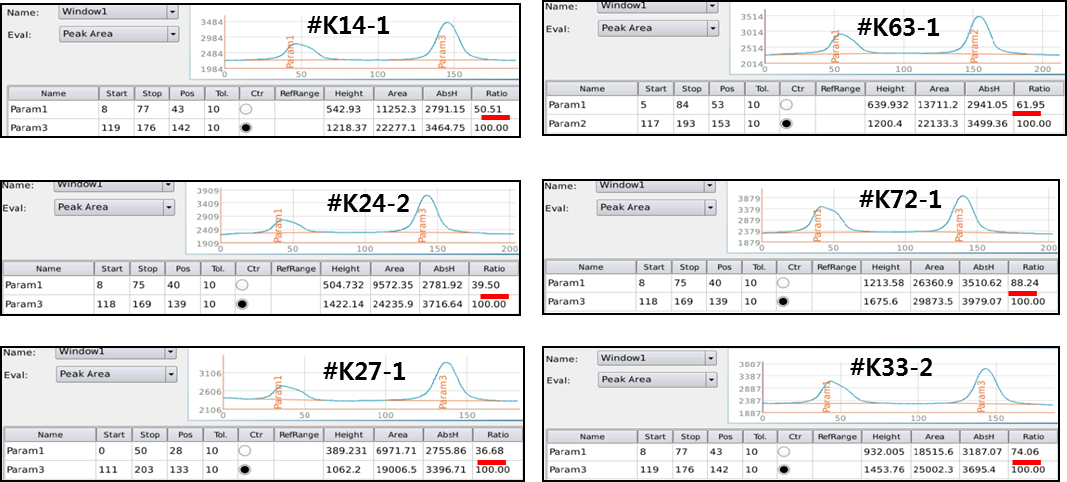
**

**Figure S5.** Raw results of rapid FICT of H1N1-positive clinical specimens. Each ratio of TL/CL was underlined with red bar (-).

**
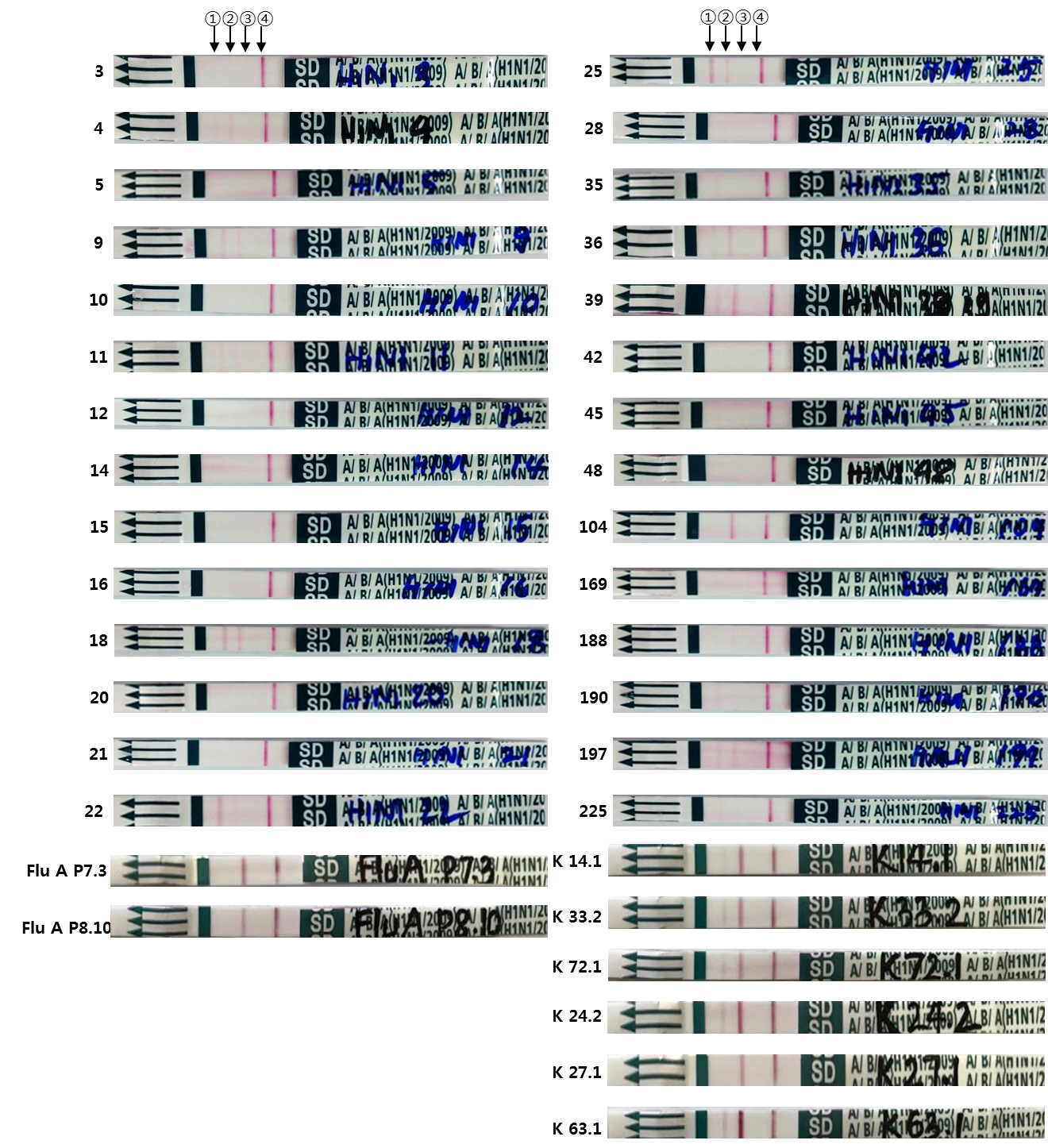
**

**Figure S6.** Raw results of RDT of clinical specimens. ①, H1N1; ②, Influenza A; ③, Influenza B; ④, Control line


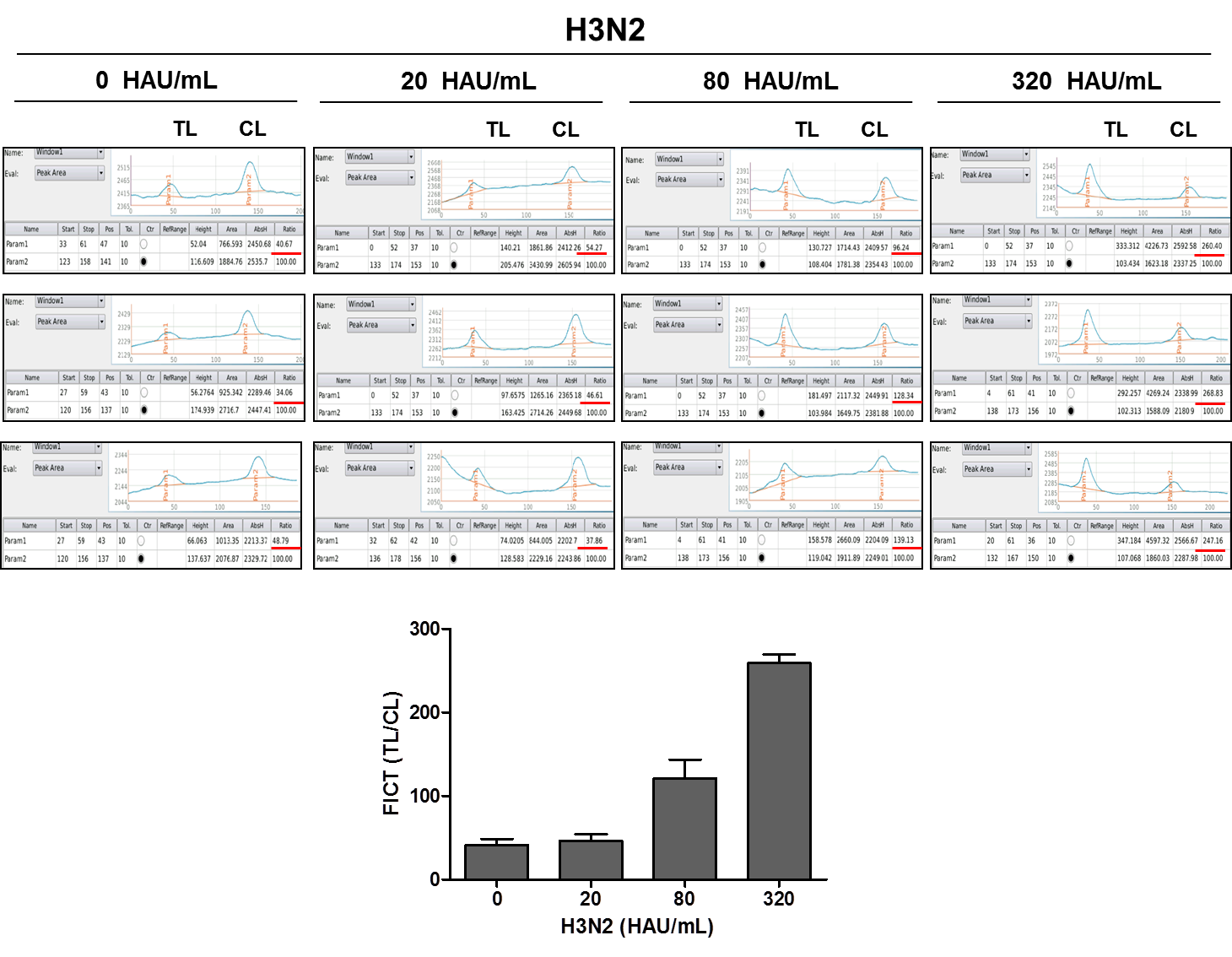


**Figure S7.** Performance of FICT to detect H3N2 virus. H3N2 virus was tested in FICT at 0, 20, 80, and 320 HAU/mL. Each ratio of TL/CL was underlined with red bar (-) and ratio of TL/CL (FICT) value was plotted in graph

**
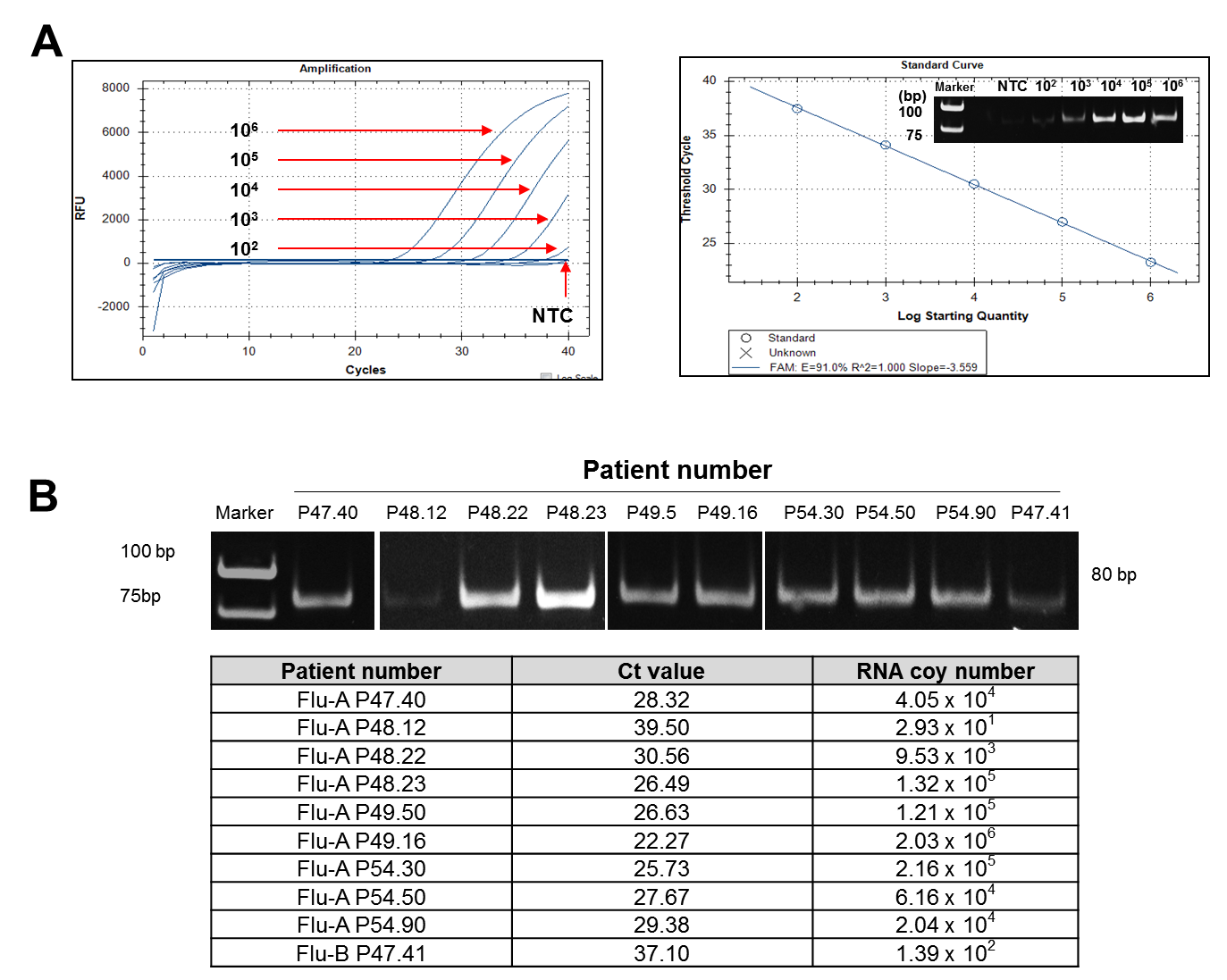
**

**Figure S8.** **rRT-PCR of H3N2-positive patients. (A)** RNA copy number and threshold cycle (Ct) of H3N2 patients was determined by rRT-PCR following previously reported protocol^1^. **(B)** RNA extract from H3N2-positive patients was conducted for rRT-PCR. Gel electrophoresis, Ct values, and RNA copy number was shown.


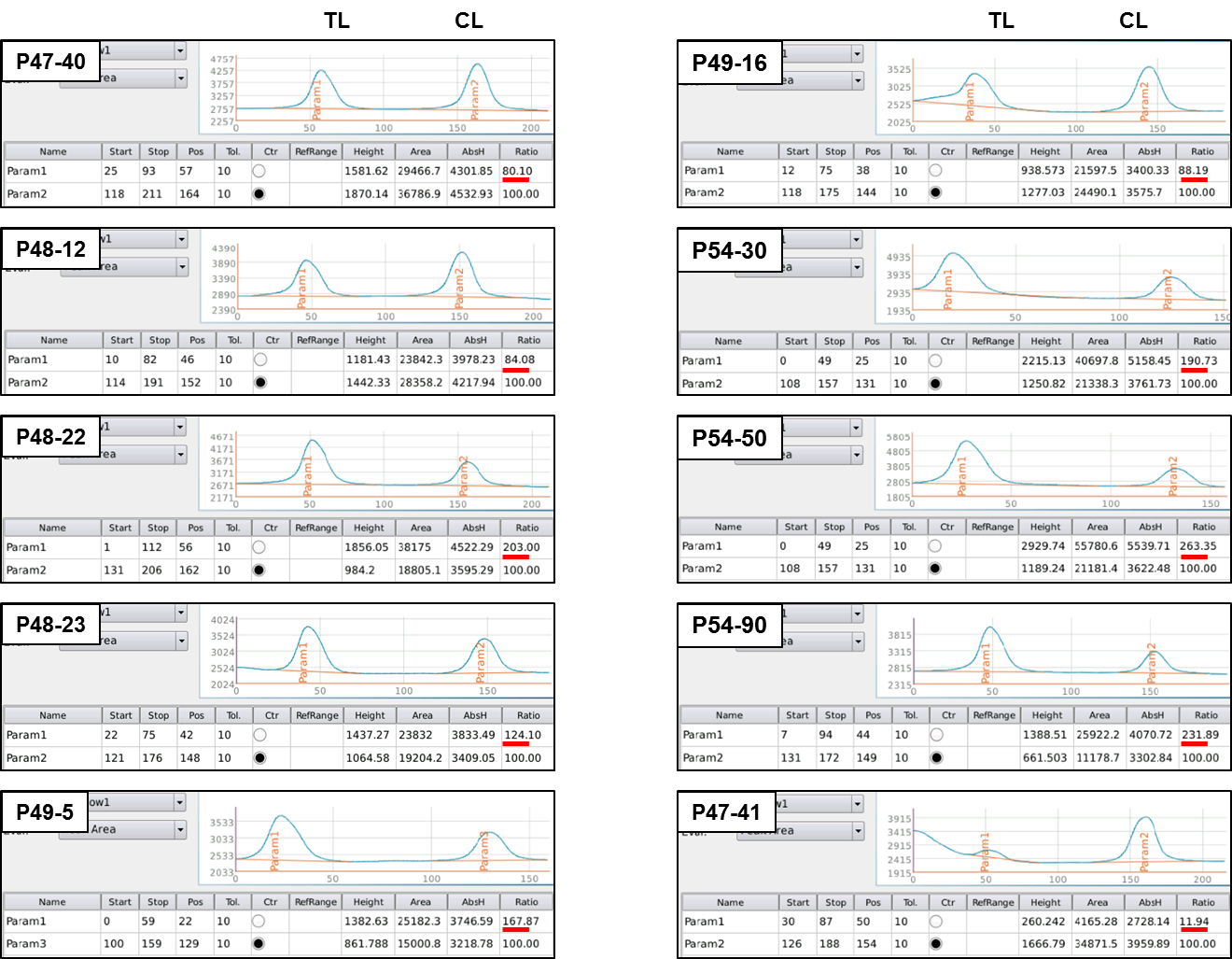


**Figure S9.** Raw results of FICT of H3N2-positive clinical specimens.

**Reference**

1. Cui D, Zhao D, Xie G, Yang X, Huo Z, Zheng S, et al. Simultaneous detection of influenza A subtypes of H3N2 virus, pandemic (H1N1) 2009 virus and reassortant avian H7N9 virus in humans by multiplex one-step real-time RT-PCR assay. Springerplus. 2016; 5: 2054.
